# Clinical Characteristics and Long-Term Mortality Rate in Female Patients with Takotsubo Syndrome Compared with Female Patients with ST-Elevation Acute Myocardial Infarction: A Retrospective Study from a Single Center

**DOI:** 10.1155/2019/9156586

**Published:** 2019-07-30

**Authors:** Laura Massobrio, Alberto Valbusa, Marina Sartini, Giovanni Meliota, Francesca Cavalla, Roberta Miceli, Giulia Vischi, Maria Luisa Cristina, Anna Maria Spagnolo, Roberto Delfino, Francesco Abbadessa, Italo Porto, Claudio Brunelli, Gian Marco Rosa

**Affiliations:** ^1^Department of Emergency Medicine, IRCCS Policlinico San Martino Genoa, Genoa, Italy; ^2^Department of Internal Medicine (DIMI) Clinic of Cardiovascular Diseases, University of Genoa, Genoa, Italy; ^3^Cardiovascular Unit, IRCCS Policlinico San Martino Genoa, Genoa, Italy; ^4^Department of Health Sciences, University of Genoa, Genoa, Italy; ^5^Department of Internal Medicine (DIMI), First Clinic of Internal Medicines, University of Genoa, Genoa, Italy; ^6^IRCCS Policlinico San Martino Genoa, Genoa, Italy

## Abstract

**Background:**

Takotsubo syndrome (TTS) is characterized by acute transient, stress-induced, left ventricular systolic dysfunction, generally presenting with apical ballooning. It can mimic an acute coronary syndrome, but with a milder increase in cardiac enzymes and without culprit coronary artery disease on angiography. Data on long-term follow-up and survival in patients with TTS, compared with patients with ST-elevation myocardial infarction (STEMI), are scarce.

**Purpose:**

To assess all-cause mortality rate and survival in a consecutive series of female patients with TTS compared with age- and sex-matched STEMI patients on long-term follow-up.

**Methods and Results:**

We collected data of 65 TTS female patients (TTS group) with a mean age of 73.42 ± 11.35 years from 2001 to 2013. Collection of follow-up information was concluded for all patients in 2016. To compare the mortality and survival of TTS patients with those of the STEMI population, we used data from our STEMI Registry, a prospective registry of 7446 STEMI patients admitted from 2001 to 2013 to our cath-lab for primary percutaneous coronary intervention (p-PCI). From the registry, we selected 104 STEMI patients (STEMI group) comparable to our TTS group in terms of age (mean age of 72.33 ± 11.92 years) and sex. On follow-up examination after a median of 1000 days, the TTS group had a lower all-cause mortality rate than the STEMI group (7.69% versus 23.08%). This difference was statistically different between the two groups (log-rank test, *p* value = 0.03).

**Conclusions:**

In our study, TTS and STEMI patients displayed a statistically significant difference in long-term survival. Specifically, the TTS group had a lower mortality rate than the STEMI group. This seems to suggest that TTS and STEMI are two different clinical entities with two different clinical outcomes.

## 1. Introduction

Takotsubo syndrome (TTS) is an acute and reversible cardiac condition characterized by transient left ventricular dysfunction [1]. This syndrome is distinct from acute myocardial infarction (AMI), although the initial presentation has similar features to ST-segment elevatiton myocardial infarction (STEMI) or non-ST-segment elevation myocardial infarction (NSTEMI).

Owing to these similarities between AMI and TTS in clinical presentation, in the earliest phase, therapy is the same consisting of both antithrombotic and heart failure drugs [2] and early and aggressive statin therapy [3, 4].

Patients with TTS have typical features (Mayo Clinic criteria) that must be identified in order to confirm the diagnosis. Recently, these features have been amended and extended (see Section 2) [5].

This clinical condition predominantly affects postmenopausal women [1]; therefore, these demographic features are not a mandatory part of the proposed diagnostic criteria.

In the literature, information concerning the long-term follow-up of patients with TTS is controversial: once believed to be a more benign condition compared with AMI [6], in recent studies, TTS appears to be burdened by significant mortality and morbidity [7, 8].

Our purpose was to assess rates of all-cause mortality and survival in a series of female patients with TTS compared with age- and sex-matched STEMI patients on long-term follow-up.

## 2. Methods

This was an observational, retrospective, single-center study. We enrolled 65 Takotsubo syndrome (TTS) female patients (TTS group) with a mean age of 73.42 ± 11.35 years, admitted to the Cardiology Division of San Martino Policlinic Hospital in Genoa, Italy, from 2001 until 2013. Clinical data regarding the history (coronary risk factors, comorbidities, and possible emotional triggers), physical examination, serum troponin I level, 12-lead electrocardiogram, echocardiography, and coronary angiography with LV angiography were collected for each patient. To reach a diagnosis of TTS, the criteria to be met are [5] as follows:

1. Transient regional wall motion abnormalities of the left or right ventricle preceded by a stress trigger. The regional wall motion abnormalities usually extend beyond a single epicardial vascular territory.
2. The absence of culprit atherosclerotic coronary artery disease or other pathological conditions to explain the pattern of temporary ventricular dysfunction observed (e.g., viral myocarditis).
3. New and reversible electrocardiography abnormalities during the acute phase, up to three months.
4. Elevated serum natriuretic peptide during the acute phase.
5. Positive, but relatively small, elevation in cardiac troponin.
6. Recovery of ventricular systolic function at follow-up.

In the TTS group, criteria for enrollment in the study cohort were as follows:

1. Admission for chest pain or equivalent symptoms, with or without ischemic electrocardiographic changes
2. Absence of obstructive epicardial coronary artery disease on the urgent coronary angiography performed
3. Evidence of transient apical or midventricular ballooning on ventriculography or on echocardiography
4. Absence of other cardiac conditions

For what regards the occurrence of complications such as acute heart failure, we referred to the following definition for “acute heart failure”: acute heart failure is a rapid onset or worsening of symptoms and/or signs of heart failure.

Enrollment of patients was concluded in 2013. Collection of follow-up information, to obtain long-term data on mortality and survival, was concluded for all patients in 2016 by telephone interview.

All patients were treated according to the current guidelines for the acute coronary syndrome and underwent echocardiographic follow-up examination to assess recovery of left ventricular function.

To compare survival in TTS patients with that of the STEMI population, we used our STEMI Registry, a prospective registry with 7446 patients admitted from 2001 until 2013 to our cath-lab for primary percutaneous coronary intervention (p-PCI). From the registry, we selected 104 STEMI patients (STEMI group) comparable to our TTS group in terms of age and sex. Data were collected by using Excel databases and processed by means of STATA/SE14 software (StataCorp, Texas, USA). Descriptive analyses were conducted initially.

Regarding long-term follow-up, statistical analysis was performed by means of a log-rank test to verify whether the differences that emerged on Kaplan–Meier survival analysis were significant; the Kruskal–Wallis test and the chi-square test were used to detect any differences between the parameters collected.

## 3. Results

We enrolled 65 Takotsubo syndrome (TTS) female patients (TTS group) with a mean age of 73.42 ± 11.35 years, from 2001 until 2013. All patients presented the “typical” form of the syndrome characterized by “apical ballooning.”

To compare survival in TTS and STEMI patients, we selected 104 STEMI patients (STEMI group) from the STEMI Registry; these had been admitted from 2001 until 2013 to our cath-lab for primary PCI and were comparable to our TTS group in terms of age (72.33 ± 11.92 years) and sex.

### 3.1. Clinical Characteristics of TTS Patients

In the TTS group, arterial hypertension and dyslipidemia were the most frequent cardiovascular risk factors (66.15% and 41.54%, respectively); 93.75% of patients were postmenopausal. Regarding comorbidities, 23% of patients presented depression disorders, 10.77% hypothyroidism, and 7.70% hyperthyroidism; the most frequent stress triggers were of an emotional nature (49.23%) (Table 1). Patients were referred to our department because of chest pain (44.62%), dyspnea (7.70%), presyncope/syncope (6.15%), sickness/vomiting (3.07%), or a combination of these symptoms, including tachycardia, as shown in Table 1. ECG on admission showed ST-elevation in 56.92% of patients (one of these had an ECG pattern with new-onset ST-segment elevation and right bundle branch block), T-wave inversion in 29.23%, and left bundle branch block in 4.61%. Only one patient presented a complete atrioventricular block (Table 1). Mean troponin peak value was 5.34 ± 5.42 *μ*g/L (Table 1). During the hospital stay, one patient (1.54%) died, one was resuscitated from cardiac arrest with secondary return of spontaneous circulation, 5 (7.69%) patients presented femoral access complications, such as hematoma or pseudoaneurysm, 2 (3.07%) patients developed hemorrhagic shock or severe anemia, 3 (4.61%) patients developed echocardiographic left ventricular thrombosis, 2 (3.07%) patients developed complete atrioventricular block, and 5 (7.69%) patients developed acute heart failure (Table 2).

**Figure 1:**
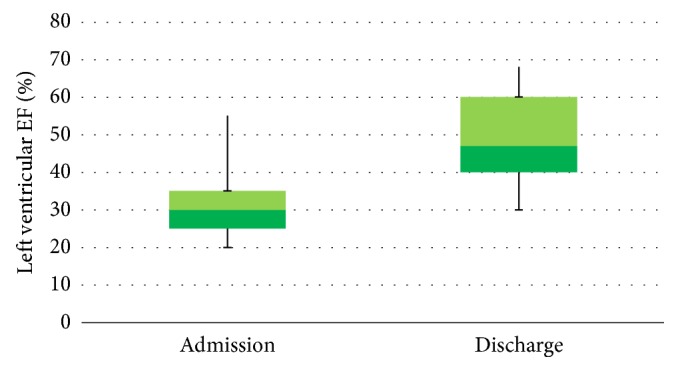
Left ventricular ejection fraction (%) on admission and on discharge in the TTS group.

On admission, the mean left ejection fraction was 41.03 ± 8.50%, while on discharge it was seen to have increased to 48.80 ± 10.75% (Table 1; Figure 1). At discharge on echocardiographic control, wall motion abnormalities had disappeared and recovery of left ejection fraction was ascertained in all patients.

During the period of follow-up, 3% of patients developed a recurrence of TTS, with secondary re-recovery of ventricular systolic function on echocardiographic examination, and 7.69% of patients died (five subjects).

Table 3 shows the main hospital admission characteristics of TTS patients who died during follow-up.

### 3.2. Clinical Differences with STEMI Patients

Concerning the difference between the TTS group and the STEMI group, the characteristics of the patients are shown in Table 4. Mean age was similar in both groups. All patients were women. Smoking and diabetes were less common in the TTS group (7.70% vs. 20.19%, *p* value < 0.01; 7.70% vs. 27.18%, *p* value = 0.001). In the TTS group, no patients had a history of previous acute myocardial infarction (0.0% vs. 9.80%, *p* value = 0.001).

**Figure 2:**
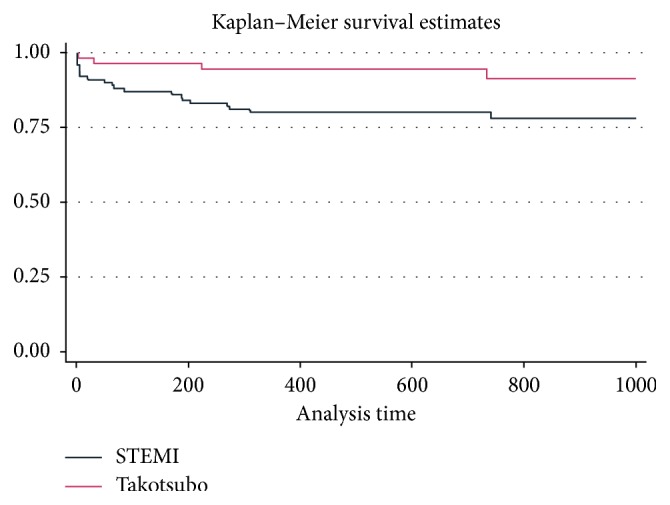
Kaplan–Meier survival estimates 1000 days TTS and STEMI group all-cause mortality.

With regard to long-term follow-up, over a median follow-up of 1000 days, the TTS group had a lower mortality rate than the STEMI group (7.69% versus 23.08%). The all-cause mortality rate over 1000 days was statistically different between the two groups (log-rank test, *p* value = 0.03). The causes of death in the TTS group were cerebral hemorrhage in one patient, sepsis in one patient, and unknown causes for the other three (two of these were over 80 years old) (Figure 2).

## 4. Discussion

Takotsubo syndrome (TTS) is a form of acute left ventricular dysfunction that mimics acute myocardial infarction in terms of both symptoms and electrocardiographic findings but without culprit coronary artery disease on angiography [1].

It has been proposed that an interplay between triggers (emotional, physical, or iatrogenic stressors) [9, 10], pathogenic mechanisms (increased catecholamine levels) [11], and predisposing factors (sex, age, and postmenopausal period) [12] could contribute through a complex interaction to cause TTS, with a variable influence on the different patients. In this context, it was suggested that also some anatomic variants of coronary vessels could have a role, through different mechanisms, in the pathogenesis of TTS [13].

Catecholamine values were not measured in our study. However, 76.93% of the patients had experienced emotional or physical stress before the acute event.

A high percentage of patients were postmenopausal (93.75%) and presented a history of arterial hypertension (66.15%), confirming the important role of sex hormones and hypertension in the pathophysiology of the disease [12]. Furthermore, they also presented depression disorders (23.08%), a history of malignancy (15.38%), and dysthyroidism (18.47%). The exact role of these comorbidities is still unknown, but they may influence sympathetically mediated myocardial stunning [11].

It is difficult to estimate the frequency of occurrence of this clinical entity in the general population: Indeed, the transitory nature of clinical and instrumental presentation may underestimate the true incidence of the syndrome [14]. Nevertheless, over the years, the incidence of TTS has increased; this may be explained in part by a changing social structure and increasing psychosocial stress [15].

Several studies on a series of Asian and Western populations have suggested that TTS accounts for 1-2% of the acute coronary syndrome [16, 17].

In this regard, on admission, TTS is typically characterized by chest pain and ST-segment abnormalities in the precordial leads, elevated cardiac enzymes, and left ventricular wall motion abnormalities, which can mimic an acute myocardial infarction.

Early differential diagnosis between these two conditions is crucial, as it may prevent unnecessary treatment of TTS patients with antithrombotic drugs [18, 19].

In our study, most patients (56.92%) presented an ECG pattern with ST-segment elevation, mimicking ST-segment elevation acute myocardial infarction (STEMI); this finding proves the difficulty of recognizing, managing, and treating this cardiomyopathy in the early acute phase.

Various studies have shown a higher rate of in-hospital complications for TTS patients [20–23]. In accordance with above studies, in our study, we found a significant occurrence of in-hospital cardiac and noncardiac complications, including 1 death and 5 patients (7.7% of the overall cohort) suffering from acute heart failure. On the other hand, there is no consensus regarding the long-term prognosis of TTS patients. Indeed, there are conflicting reports and data regarding the long-term survival of patients after the initial episode of TTS. Specifically, in the prospective SWEDEHEART Registry, 3-year all-cause mortality in the Takotsubo syndrome group was similar to that of NSTEMI and STEMI controls [18].

In GEIST Registry, Stiermaier et al. reported that all-cause mortality rates in TTS patients were considerably higher than previously expected and long-term mortality exceeded that of patients presenting with STEMI [24]. Male sex, diabetes mellitus, Killip class 3/4 on admission, and secondary TTS were predictive factors of mortality [24]. A large, recently published metaregression analysis found a nonnegligible rate of long-term mortality in TTS; furthermore, it showed that physical stressors, along with older age and atypical variants, were associated with worse prognosis [8].

We found a significantly lower all-cause mortality for TTS patients compared with STEMI controls in our population. Notably, however, our TTS patients were all women (100%), few had diabetes (8%), few were in class Killip class 3/4 during hospitalization (8%), while the percentage of emotional stressor triggers was high (49%). These epidemiological differences may, at least in part, account for our results. On the other hand, in an interesting study, Looi et al. have questioned, due to methodological issues, the worse outcome of TTS patients compared with ACS cohorts [25]. Indeed, in this study, it was found a higher rate of mortality when comparing TTS patients with age- and gender-matched community controls with no known cardiovascular diseases, but a better outcome when comparing TTS patients with a cohort of hospitalized ACS patients [25].

Furthermore, both Looi et al. [25] and Almendro Delia et al. [26], in their studies, have shown that long-term mortality in TTS patients is mainly driven by noncardiovascular causes. On the contrary, while the residual ischemic burden of ACS is well known and significantly influences prognosis in STEMI and NSTEMI patients [27], the reversible nature of myocardial alterations in TTS may probably affect the long-term outcome at a lesser extent. In this context, the role of comorbidities, both cardiac and noncardiac, may play a role and still needs to be fully assessed [1, 28]. It appears however reasonable that definite subsets of TTS patients may present different outcomes, at both short term and long term, as it has been shown for the diverse variants [29] and diverse stressors [8]. Again, the female gender of our TTS cohort may have been a protective factor responsible for our findings [30].

## 5. Conclusions

Our study suggests a benign course of patients with TTS, emphasized by the fact that recurrence episodes of TTS are infrequent. We supposed that underlying noncardiac diseases are the only independent predictors of long-term mortality of these patients. Takotsubo syndrome (TTS) is a reversible acute heart failure syndrome, but current knowledge to guide optimal clinical management is insufficient and data on the long-term prognosis are limited and often conflicting [2]. To date, clinical reports are mostly based on relatively small cohorts and case series. Therefore, randomized controlled trials are needed to identify optimal management strategies for Takotsubo syndrome.

## Figures and Tables

**Table 1:** TTS patient characteristics.

Clinical/ECG/echo characteristics	TTS group *N* = 65
Age (years)	73.42 ± 11.35
Risk factors for CAD	
Family history of CAD	9 (13.85)
Hypertension	43 (66.15)
Hyperlipidemia	27 (41.54)
Diabetes	5 (7.70)
Smoking	5 (7.70)
Postmenopausal period	61 (93.75)
Recent stressful events	
Physical stressful events	18 (27.70)
Emotional stressful events	32 (49.23)
Unknown	15 (23.07)
Depression disorders	15 (23.08)
History of malignancy	10 (15.38)
Dysthyroidism	
Hypothyroidism	7 (10.77)
Hyperthyroidism	5 (7.70)
Type of symptoms	
Chest pain alone	29 (44.62)
Dyspnea alone	5 (7.70)
Chest pain and dyspnea	13 (20)
Presyncope/syncope	4 (6.15)
Dyspnea and syncope	1 (1.54)
Chest pain and tachycardia	1 (1.54)
Dyspnea and tachycardia	1 (1.54)
Syncope and tachycardia	1 (1.54)
Chest pain and sickness	3 (4.61)
Chest pain dyspnea and sickness	4 (6.15)
Chest pain syncope and sickness	1 (1.54)
Sickness/vomiting	2 (3.07)
Mean left ventricular ejection fraction at admission (%)	41.03 ± 8.50 (20–65)
Mean left ventricular ejection fraction at discharge (%)	48.80 ± 10.75 (30–68)
Troponin I peak value (*μ*g/L)	5.34 ± 5.42 (0.05–32.87)
Electrocardiographic findings on admission	
ST-segment elevation^*∗*^	37 (56.92)
T-wave inversion	19 (29.23)
Left bundle branch block (new onset)	3 (4.61)
Complete atrioventricular block	1 (1.54)
Normal ECG pattern	5 (7.70)

ECG: electrocardiogram; echo: echocardiographic; CAD: coronary artery disease. ^*∗*^One patient presented ST-segment elevation + right bundle branch block of new onset.

**Table 2:** TTS clinical complications during hospital stay.

TTS in-hospital complications	*N* = 65
Death	1 (1.54)
Cardiac arrest with secondary ROSC	1 (1.54)
Femoral hematoma or pseudoaneurysm	5 (7.70)
Hemorrhagic shock or severe anemia	2 (3.07)
Echocardiographic left ventricle thrombosis	3 (4.61)
Total atrial-ventricular block	2 (3.07)
Acute heart failure	5 (7.70)
Atrial fibrillation	1 (1.54)
Nonsustained ventricular tachycardia	1 (1.54)
Hypocalcemia/hyponatremia	2 (3.07)

ROSC: return of spontaneous circulation.

**Table 3:** The main hospital admission characteristics of TTS patients who died during follow-up.

	Patient 1	Patient 2	Patient 3	Patient 4	Patient 5
Age	81	75	71	79	91
Left ventricular ejection fraction at discharge (%)	35	50	42	30	30
Ballooning type	Apical	Apical	Apical	Apical	Apical
Stressor	Ischemic stroke		Rhabdomyolysis	Neurosurgical intervention	Quarrel
Symptoms	Dyspnoea and Syncope	Dyspnoea alone	Syncope alone	Chest pain alone	Chest pain alone
ECG	Normal	T-wave inversion (leads V2–V6)	ST-elevation in anterior leads	ST-elevation in anterior leads	ST-elevation in inferolateral leads

**Table 4:** Clinical characteristics of TTS and STEMI groups.

Variable	TTS group *N* = 65	STEMI group *N* = 104	*p* value
Female	65 (100)	104 (100)	
Age (mean ± SD)	73.42 ± 11.35	72.33 ± 11.92	NS
Smoking	5 (7.70)	21 (20.19)^*∗*^	<0.01
Diabetes	5 (7.70)	17 (27.18)	=0.001
Hypertension	43 (66.15)	66 (63.64)	NS
Hyperlipidemia	27 (41.54)	44 (42.42)	NS
Previous AMI	0	10 (9.80)	=0.001

AMI: acute myocardial infarction; CABG: coronary artery bypass graft; PCI: percutaneous coronary intervention; NS: not significant. ^*∗*^Anamnestic data missing in eight (12.50%) patients.

## Data Availability

The data used to support the findings of this study are present in the registry of patients of San Martino Policlinic Hospital.

## Additional Points

*Study Limitations*. This study has several limitations: Firstly, it was an observational, retrospective, single-center study. Secondly, we did not have data on cause-specific mortality, as the telephone interviews used did not allow us to obtain more specific information. Furthermore, the exclusion of patients with missing data might have biased the results. Thirdly, the matching procedure of TTS and STEMI should result in an equal number of patients for each group, while this study included 65 TTS and 104 STEMI patients. Fourthly, data on hemorrhagic complications were influenced by the fact that the first coronary angiographies in our patients were performed through femoral artery access. Finally, that exclusion of atypical ballooning pattern may have reduced the generalizability of the study results.
